# Volumetric Modulated Arc Therapy for Spine Radiosurgery: Superior Treatment Planning and Delivery Compared to Static Beam Intensity Modulated Radiotherapy

**DOI:** 10.1155/2016/6805979

**Published:** 2016-01-13

**Authors:** Leor Zach, Lev Tsvang, Dror Alezra, Maoz Ben Ayun, Ran Harel

**Affiliations:** ^1^Radiation Oncology unit, Oncology Institute, Sheba Medical Center, Ramat Gan, Israel; ^2^Sheba Medical Center, Israel; ^3^Stereotactic Radiosurgery Unit, Talpiot Medical Leadership Program, Department of Neurosurgery, Sheba Medical Center, Ramat Gan, Israel; ^4^Spine Surgery Unit, Talpiot Medical Leadership Program, Department of Neurosurgery, Sheba Medical Center, Ramat Gan, Israel

## Abstract

*Purpose*. Spine stereotactic radiosurgery (SRS) delivers an accurate and efficient high radiation dose to vertebral metastases in 1–5 fractions. We aimed to compare volumetric modulated arc therapy (VMAT) to static beam intensity modulated radiotherapy (IMRT) for spine SRS.* Methods and Materials*. Ten spine lesions of previously treated SRS patients were planned retrospectively using both IMRT and VMAT with a prescribed dose of 16 Gy to 100% of the planning target volume (PTV). The plans were compared for conformity, homogeneity, treatment delivery time, and safety (spinal cord dose).* Results*. All evaluated parameters favored the VMAT plan over the IMRT plans. *D*_min_ in the IMRT was significantly lower than in the VMAT plan (7.65 Gy/10.88 Gy, *p* < 0.001), the Dice Similarity Coefficient (DSC) was found to be significantly better for the VMAT plans compared to the IMRT plans (0.77/0.58, resp., *p*  value < 0.01), and an almost 50% reduction in the net treatment time was calculated for the VMAT compared to the IMRT plans (6.73 min/12.96 min, *p* < 0.001).* Conclusions*. In our report, VMAT provides better conformity, homogeneity, and safety profile. The shorter treatment time is a major advantage and not only provides convenience to the painful patient but also contributes to the precision of this high dose radiation therapy.

## 1. Introduction

**Figure 1:**
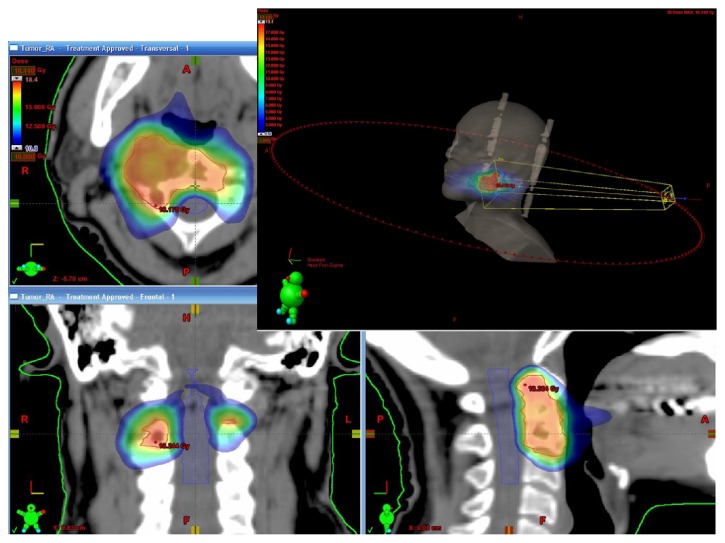
VMAT spinal SRS treatment plan. An example of a volumetric modulated arc therapy (VMAT) stereotactic radiosurgery (SRS) plan to C2 spinal vertebrae. The right side demonstrates the 360° arc therapy concept. The image demonstrates the highly conformal and homogenic dose to the treated volume with a very rapid decrease of the dose in the spinal canal, which allows highly effective and safe radiation treatment.

Spinal metastases are the most frequent spine tumors and can significantly impact a patient's quality of life as a result of disabling pain, fractures, or paralysis due to spinal cord compression [1–3]. Fractionated radiation therapy is considered the treatment of choice for most cases [1]. Spine stereotactic radiosurgery (SRS) (also referred to as stereotactic body radiotherapy or SBRT) is a novel treatment modality that delivers an accurate and conformal high radiation dose to the tumor in 1–5 sessions, with steep fall-off dose gradients that protect adjacent normal structures [4–8]. High resolution computed tomography (CT) and magnetic resonance imaging (MRI) images are used for treatment planning. The routine method for treatment planning and delivery is intensity modulated radiotherapy (IMRT). Multiple (usually static) beams of radiation are directed from various 7–10 different angles. A computerized algorithm is used to calculate the motion of the multileaf collimator (MLC) for each field in order to achieve the best dose distribution according to a predetermined dose constraints [3, 6, 8–10]. Treatment must be administered with very high precision to achieve full coverage of the tumor, while avoiding radiation of the spinal cord and other adjacent organs at risk (OAR). Precise radiation delivery requires patients to be motionless during treatment, and they are immobilized in the same device used for pretreatment CT and sometimes the MRI scans. Patient position is continuously monitored and corrected with on board imaging systems and a robotic coach [6, 10]. volumetric modulated arc therapy (VMAT) is a radiation treatment method combining IMRT planning algorithms which relies on dynamic arcs for treatment delivery [11, 12] and is currently used by the authors for spine SRS (Figure 1). In the current report we aimed to compare the planning and the delivery of spine SRS treatment using static IMRT fields and VMAT. The retrospective analysis was approved by the local Ethics Committee.

## 2. Methods and Materials

 Ten spine lesions of previously treated SRS patients were planned retrospectively using both
  - IMRT (iPLAN, BrainLab, Germany) with 7 coplanar static fields.
  - VMAT (RapidArc, Eclipse, Varian, USA) using 2-3 dynamic arcs.
 The prescribed dose was 16 Gy to 100% of the planning target volume (PTV).
 The highest priority OAR was the spinal cord while the other OAR's (such as the esophagus, lungs, kidneys, and swallowing apparatus) dose was monitored during plan evaluation but without prioritizing them in the planning algorithm [13].

The plans were compared for conformity, homogeneity, treatment delivery time, and safety (spinal cord dose).

### 2.1. Conformity and Homogeneity

Conformity is a measure of the dose coverage to the PTV. There are several indexes that are aimed to measure the conformity of a radiation plan. The closer the value of a conformity index (CI) to 1, the better the coverage of the target volume by a certain dose. We calculated the CI for each plan, calculated the absolute difference between each value to 1 (since the CI might get values above and below 1), and then compared the IMRT and VMAT plans. We chose to use 2 indexes in our comparison:

(1) The RTOG formula [14] is

(1) $$\begin{array}{c} CI=\frac{V_{D\%}}{V_{PTV}}. \end{array}$$

CI is the conformity index,  PTV is the planned target volume, and *V*_*D*%_ is the volume within the PTV, which is treated by at least *D*% of the prescribed dose.

We looked at *V*_98%_, *V*_95%_ corresponding with the irradiated volume receiving 98% and 95% of the prescribed dose.

(2) Dice Similarity Coefficient (DSC) [15] is the ratio between *V*_98%_ intersection with the *V*_PTV_ and* V*_98%_ union with *V*_PTV_:

(2) $$\begin{array}{c} DSC=\frac{{TV}_{98\%}\cap V_{PTV}}{{TV}_{98\%}\cup V_{PTV}}. \end{array}$$

TV_98%_ is the total volume (in and out of the PTV) receiving at least 98% of the prescribed dose.

**Figure 2:**
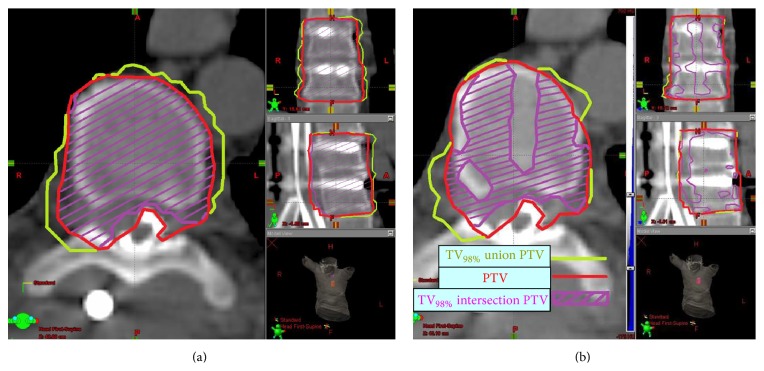
A graphic comparison of Dice Similarity Coefficient (DSC) in VMAT (a) and IMRT (b) plans. The DSC is the ratio between the *V*_98%_ intersection with *V*_PTV_ (purple) to the *V*_98%_ union with *V*_PTV_ (yellow). The VMAT allows a much conformal plan (a) where 98% of the dose is given to almost all of the PTV and does not leak outside the PTV compared to the IMRT plan.

The DSC contributes an added value compared to the CI by investigating more thoroughly the relationship between the two volumes (Figure 2).

Homogeneity of a radiotherapy plan is a measure of similarity of the dose within the target volume. The plan is considered to be better when the homogeneity index (HI) is smaller and closer to 0 [16]. Consider

(3) $$\begin{array}{c} HI=\frac{\left(D_{2\%}-D_{98\%}\right)}{D_{50\%}}. \end{array}$$

 
*D*_2%_ is the dose to at least 2% of the target volume, *D*_98%_ is the dose to at least 98% of the target volume, and *D*_50%_ is the dose to at least 50% of the target volume.

### 2.2. Treatment Time

Since the patients were already treated and it is impossible to treat a single patient with both IMRT and VMAT, there is no direct way to compare the “on board time” for the execution of each plan. We compared the monitor units needed in each plan to compute the net irradiated time.

### 2.3. Spinal Cord Dose

The volume of spinal cord contoured for each plan was 6 mm above and below the treated vertebrae. We compared *D*_max_ and *V*_10 Gy_ (the volume of the spinal cord exposed to at least 10 Gy) since these have been found to be predictive to radiation myelopathy [8–10, 17].

The iPLAN (BrainLab, Germany) algorithm of the IMRT puts the spinal cord *V*_10 Gy_ constraint in the highest priority and compromises the dose to the PTV when needed. To allow a valid and meaningful (although theoretical) comparison of *V*_10 Gy_ between the two radiation plans that were generated with different planning software and algorithms, we transferred the IMRT plans from the iPLAN (Brainlab, Germany) to the Eclipse (Varian, USA) system and normalized it to the VMAT plan's 95% isodose line (a dose which was considered acceptable for PTV coverage).

### 2.4. Statistical Considerations

All the above values were calculated for each treatment plan. We calculated the average value for each plan and the standard deviation. We then used two-tailed type 1 Student's *t*-test to compare the average values and compute the significance *p* value for the comparisons between the IMRT and the VMAT plans.

## 3. Results

Ten lesions from 6 patients were analyzed (3 males and 3 females). All patients had metastatic solid tumors to the spinal vertebrae (breast cancer (3), soft tissue sarcoma (1), adrenocortical carcinoma (1), and renal cell carcinoma (1)) indicated for spinal radiosurgery either due to tumor recurrence after standard radiotherapy, oligometastatic disease, or radioresistant histology.

The treated lesions involved cervical, thoracic, and lumbar areas of the spine.

The PTV mean volume was 67.87 cm^3^ (range 38–141 cm^3^) and the mean irradiated spinal cord volume was 4.55 cm^3^ (range 0.8–18.7 cm^3^).

Overall the VMAT plans were superior to the IMRT plan in almost every aspect of our comparison.

### 3.1. Conformity and Homogeneity

**Figure 3:**
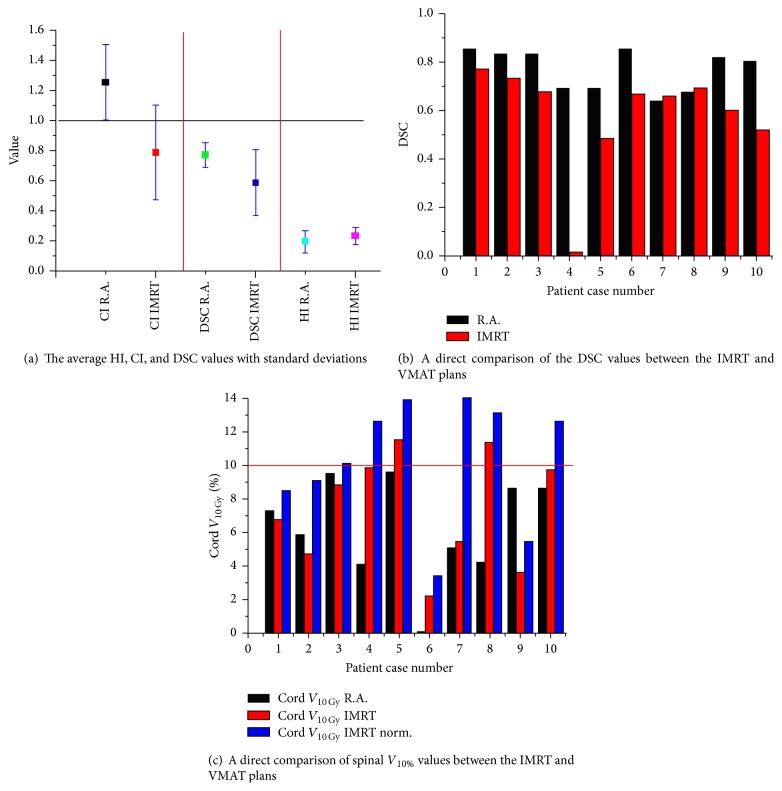
Conformity, homogeneity, and safety comparison between the VMAT (black) and IMRT (red) plans.

All the calculated indexes favored the VMAT plan over the IMRT plans (Figure 3(a)). While there was no significant difference in the *D*_max_ and the HI between the IMRT and the VMAT plans, the *D*_2%_, *D*_min_, *D*_50%_, and *D*_98%_ to the PTV were significantly lower in the IMRT plan compared to the VMAT plan (*p* < 0.01) suggesting a favorable dose volume histogram (DVH) in the VMAT plans (Table 1).

The conformity indexes comparisons on the other hand generated intriguing results: While CI_95%_ and CI_98%_ of the IMRT and VMAT were not significantly different, the more sensitive measure of conformity, the Dice Similarity Coefficient (DSC) was found to be significantly better for the VMAT plans compared to the IMRT plans (*p*  value < 0.01) (Table 1, Figure 3(b)).

### 3.2. Treatment Time

The mean number of monitor units in the VMAT plans was significantly lower in the VMAT compared to the IMRT plans (6313/10474, resp., *p* < 0.01), corresponding with an almost 50% reduction in the net treatment time for the VMAT compared to the IMRT plans (6.73 min/12.96 min, resp., *p* < 0.001).

### 3.3. Spinal Cord Dose

The mean spinal cord dose was significantly lower in the IMRT plans compared to the VMAT plans (5.19 Gy/7.03 Gy, resp., *p* < 0.001).

Nevertheless, although *V*_10 Gy_ was not significantly different between the IMRT and VMAT plan in a direct comparison, a significantly higher volume was found after the normalization in the IMRT plan (10.4%/5.75%, resp., *p* < 0.01). Furthermore, after the normalization to ensure 95% of the dose PTV coverage, the IMRT plan did not meet the predefined constraint of *V*_10 Gy_ < 10% in more than half (50%) of the cases.

Average *D*_max_ to the spinal cord was significantly higher in the IMRT compared to the VMAT plans (13.73/12 Gy, resp., *p* < 0.001) and a dose of >14 Gy (prescribed *D*_max_ constraint to the spinal cord) was found in some of the IMRT plans but in none of the VMAT plans (Figure 3(c)).

## 4. Discussion

Patients with spinal cord metastases suffer from debilitating pain, neurologic deficits, and secondary functional deterioration in Karnofsky performance status (KPS), ambulation, and quality of life (QOL). Spine SRS provide state-of-the-art rapid relief with long term local control with functional and QOL improvement [5–7, 18, 19].

Previous publications have tried to compare VMAT technology to IMRT for spine SRS planning with conflicting results [11, 12].

Wu et al. [11] tried to compare 1- and 2-arc VMAT plans to IMRT (all patients were previously treated with the IMRT plans) demonstrating better conformity in the 2-arc VMAT plan but spinal cord sparing was inferior in the VMAT plans (the difference was not significant in the 2-arc plans). Marchand et al. [12] reported a comparison of IMRT with single-arc VMAT in 15 previously treated patients. The single-arc VMAT for spine SBRT improved delivery efficiency while maintaining target coverage and OAR sparing compared to IMRT [12].

We aimed to describe our experience with the first 10 lesions while explaining our clinical and dosimetric arguments to move forward from IMRT to VMAT planning.

In our comparison, there was no significant difference in the mean homogeneity of the dose between the plans but we recognized lower *D*_2%_ and *D*_min_ in the IMRT plans. The clinical significance of cold spots within the target volume can be judged only with a prospective follow-up of patients and recurrence rate, which is beyond the scope of this report.

Our use of two separate CI formulas yielded intriguing results regarding the concordance between the target volume and the actual treated volume. The DSC which is more complicated to calculate proved to be more sensitive and perhaps more reliable than the standard CI and should be adopted in future dosimetric comparisons and investigations.

The safety of an SRS plan is of immeasurable importance and the spinal cord dose is the whole mark of this treatment. The fact that to allow comparable PTV dose coverage (using normalization of the IMRT plans to the VMAT plans) the IMRT demonstrated higher cord point doses and in some of the cases failed to achieve the predefined constraints is of major concern.

Our results are in concordance with previous reports where treatment duration was significantly shorter with the VMAT plans [11, 12]. Painful patients sometimes can not complete a treatment session even with heavy analgesics. The pain may induce patients' movement which reduces the treatment precision, a problem that can not be underestimated in spinal cord radiosurgery.

## 5. Conclusions

The use of VMAT as a superior planning and treatment delivery method for spinal cord radiosurgery has been suggested before. We believe that our report adds dosimetric evidence that the VMAT provides better conformity, better homogeneity, and a better safety profile. We are collecting prospective clinical data regarding the actual control and complication rates with VMAT spine radiosurgery to enhance these conclusions. We also suggest that the shorter treatment time is a major advantage and not only provides convenience to the painful patient but contributes to the precision of this high dose radiation therapy.

## Figures and Tables

**Table 1:** Dose volume histogram (DVH) values, homogeneity index (HI), and conformity indexes (CI and DSC) values.

Plan	Value	*D*_98 (Gy)_	*D*_95 (Gy)_	*D*_2 (Gy)_	*D*_50 (Gy)_	*D*_max (Gy)_	*D*_min (Gy)_	HI	1 − CI_98_	1 − DSC
Mean (STD)	IMRT	13.54 (0.9)	14.65 (0.5)	16.92 (1.2)	16.02 (0.3)	18.56 (0.8)	7.65 (1.6)	0.21 (0.1)	0.38 (0.4)	0.42 (0.2)
	RA	14.74 (0.9)	15.73 (0.5)	18.09 (0.7)	17.36 (0.5)	18.79 (0.8)	10.88 (2)	0.19 (0.1)	0.31 (0.3)	0.23 (0.1)

The optimal conformity index value is one but may be above or below this value. We calculated and show the absolute value of the mean differences between the CI/DSC and 1.
